# Transection of the cervical sympathetic trunk ameliorates pulmonary arterial hypertension by reprogramming transcription factor networks and alternative splicing in rat model

**DOI:** 10.3389/fcvm.2026.1967847

**Published:** 2026-10-28

**Authors:** Yu Hao, Peipei Li, Hua Cheng, Ning Yan, Hui Zhang, Xueping Ma

**Affiliations:** 1 Department of Cardiology, General Hospital of Ningxia Medical University, Yinchuan, Ningxia, China; 2 Department of Anesthesiology, General Hospital of Ningxia Medical University, Yinchuan, Ningxia, China

**Keywords:** alternative splicing, calcium signaling, PAH, TCST, transcription factor network

## Abstract

**Background:**

Transection of the cervical sympathetic trunk (TCST) ameliorates pulmonary arterial hypertension (PAH), but the molecular mechanisms remain unclear. This study aimed to elucidate these mechanisms in monocrotaline (MCT)-induced PAH rats through transcriptomic profiling.

**Methods:**

PAH rats were divided into control, PAH, PAH + sham, and PAH + TCST groups. Therapeutic effects were evaluated by hemodynamics and histology. RNA-seq was performed on lung tissues to identify differentially expressed genes (DEGs), followed by transcription factor (TF) network analysis and alternative splicing analysis, with validation by qPCR and ELISA.

**Results:**

TCST significantly reduced mPAP, RVSP, and RVHI, and attenuated vascular remodeling. RNA-seq identified 500 DEGs (61 upregulated, 439 downregulated). TF network analysis revealed that target genes of differentially expressed TFs were enriched in inflammation, proliferation, and calcium signaling. Multiple PAH-associated genes (*Bmp2*, *Ptgs2*, *Cxcl13, Wnt7b*, *Tacr1*, *Adra1d*) were downregulated following TCST. Alternative splicing analysis showed that TCST modulated RNA-binding protein (RBP) expression and altered splicing patterns of PAH-relevant genes (*Aldh2*, *Il1rn*, *Foxm1*). qPCR and ELISA confirmed suppression of the *Foxa3*/*Adra1d*-*Tacr1* axis and reduced IP3 production.

**Conclusions:**

TCST exerts therapeutic effects through selective reprogramming of TF networks and regulation of RBP-mediated alternative splicing, coordinately suppressing PAH-associated pathways at transcriptional and post-transcriptional levels. These findings provide mechanistic insights and a theoretical basis for developing neuromodulatory strategies for PAH.

## 1 Introduction

Pulmonary arterial hypertension (PAH) is a progressive and heterogeneous disorder characterized by a sustained elevation in mean pulmonary arterial pressure (mPAP) > 20 mmHg at rest, arising predominantly from excessive pulmonary vasoconstriction and maladaptive structural remodeling of the pulmonary vasculature (1, 2). This hemodynamic abnormality arises predominantly from excessive pulmonary vasoconstriction and maladaptive structural remodeling of the pulmonary vasculature. This pathological process leads to increased pulmonary vascular resistance (PVR), right ventricular (RV) afterload, and ultimately RV failure and death (3, 4). Current pharmacological management primarily targets three classical pathways: the prostacyclin, endothelin-1 and nitric oxide systems (5). These therapies aim to modulate pulmonary vascular tone and induce vasodilation, thereby reducing right ventricular afterload and improving cardiopulmonary hemodynamics (6). While they significantly enhance patients' quality of life and exercise capacity, their long-term survival benefits remain limited, underscoring the urgent need for novel therapeutic strategies (7).

However, research indicates that beyond conventional pathways, several abnormal signaling pathways play crucial roles in the pathogenesis of PAH. Aberrant proliferation of pulmonary arterial smooth muscle cells (PASMCs) is a hallmark of pulmonary vascular remodeling, driven by dysregulated signaling cascades including MAPK (8), PI3 K/Akt (9), and calcium-dependent pathways (10). Concurrently, inflammation has emerged as a critical contributor to PAH pathogenesis, characterized by perivascular infiltration of macrophages, T lymphocytes, and mast cells, as well as elevated levels of pro-inflammatory cytokines such as IL-1β, IL-6, and TNF-α (11, 12). These inflammatory processes interact closely with PASMC proliferation, as cytokines can directly stimulate smooth muscle cell growth and migration, while activated immune cells release growth factors that exacerbate vascular remodeling (13, 14). Notably, accumulating evidence suggests that targeting either PASMC proliferation or inflammation—or ideally both—may offer therapeutic benefit in PAH (8, 12). Consequently, a deeper investigation into the underlying pathogenic mechanisms of PAH is of critical importance, as it holds significant scientific and clinical importance for identifying novel therapeutic targets and developing effective intervention strategies.

Pulmonary vessels receive innervation from sympathetic, parasympathetic and sensory nerves. Among these, sympathetic nerves mediate vasoconstriction and elevate vascular resistance primarily via *α*-adrenergic receptors (15). Accumulating evidence over the past two decades has consistently demonstrated that PAH is frequently associated with overactivation of the sympathetic nervous system (SNS) (16–19). Elevated SNS activity in PAH patients correlates with poor prognosis (16, 20), and may even serve as an independent predictor of clinical worsening (17, 21). Interventions that mitigate sympathetic overexcitation, such as beta-blockers, can reduce cardiac load, lower blood pressure and decrease myocardial oxygen consumption, thereby conferring cardioprotective effects (22, 23). Given the recognized role of SNS activation in PAH, various strategies to attenuate this activation have been explored (15), including renal denervation (24, 25) and pulmonary artery denervation (PADN) (26–28). Although some experimental and clinical studies suggest that these approaches can reduce mPAP and slow PAH progression, their precise mechanisms remain incompletely elucidated. Moreover, radiofrequency ablation carries risks of vascular stenosis or unintended damage to parasympathetic nerves, which may compromise their safety profile (29). Therefore, there is a pressing need to develop more precise and safer methods for sympathetic intervention.

Stellate ganglion block (SGB) is a technique that modulates sympathetic nerve activity, potentially improving immune function, correcting neuroendocrine imbalances and alleviating systemic sympathetic overexcitation. In recent years, SGB has demonstrated efficacy in treating various conditions (30–32). Transection of the cervical sympathetic trunk (TCST) is considered an appropriate animal model to simulate the long-term effects of SGB (33). Preliminary studies indicate that TCST can ameliorate PAH symptoms in rats (29); however, the specific molecular mechanisms underlying this improvement remain unclear.

In this study, we established a monocrotaline (MCT)-induced rat model of PAH to systematically evaluate the effects of TCST on pulmonary arterial pressure, right ventricular function and lung tissue pathology. Furthermore, we employed whole transcriptome sequencing to analyze differentially expressed genes and their associated signaling pathways in lung tissue following TCST intervention, with a particular focus on transcription factor networks and RNA-binding protein (RBP)-mediated alternative splicing. Our aim was to elucidate the potential molecular mechanisms by which TCST alleviates PAH at both the transcriptional and post-transcriptional levels, thereby providing a theoretical foundation for developing novel PAH treatment strategies based on neuroimmunomodulation.

## 2 Materials and methods

### 2.1 Animals

Adult male Sprague-Dawley rats (weight 180–220 g, age 6–8 weeks) were obtained from Hubei BioNT Animal Center. All animals were housed under specific pathogen-free conditions with a 12 h light/dark cycle, controlled temperature (22–24 °C), and humidity (50%–60%), with free access to standard chow and water. All experimental procedures were approved by the Institutional Animal Care and Use Committee of Hubei BioNT Experimental Center (Approval No. BNB-2025-023) and conformed to the National Institutes of Health Guide for the Care and Use of Laboratory Animals.

### 2.2 MCT-induced PAH rat model and TCST surgery

Following a 7-day acclimation period, rats were randomly assigned to four groups (*n* = 8 per group): (1) Normal Control (NC) group: received a subcutaneous injection of an equal volume of phosphate-buffered saline (PBS); (2) PAH group: received a single subcutaneous injection of monocrotaline (MCT, 60 mg/kg, C101555-50, ALADDIN, China); (3) PAH + sham group: received MCT injection followed by sham surgery, which involved exposure of the cervical sympathetic trunk without ligation or transection; (4) PAH + TCST group: received MCT injection followed by transection of the cervical sympathetic trunk (TCST).

For TCST surgery, rats were anesthetized with intraperitoneal injection of pentobarbital sodium (40 mg/kg), which provided stronger anesthesia and was more convenient for subsequent surgical procedures. A midline cervical incision was made, and the left cervical sympathetic trunk was carefully isolated under a stereomicroscope. In the PAH + TCST group, the sympathetic trunk was ligated with 6-0 silk suture and transected distally to the ligation. TCST was performed on the same day (Day 7) following MCT injection, and successful denervation was confirmed by the onset of ipsilateral Horner's syndrome (ptosis, miosis, and enophthalmos) within 24 h post-surgery. All animals were sacrificed 28 days after MCT injection (i.e., 21 days after TCST, Figure 1A). Immediately after surgery, the rats were placed on a constant temperature heating pad until their righting reflex was restored and they can move autonomously. Subsequently, rats were injected with meloxicam daily for 2–3 days.

**Figure 1:**
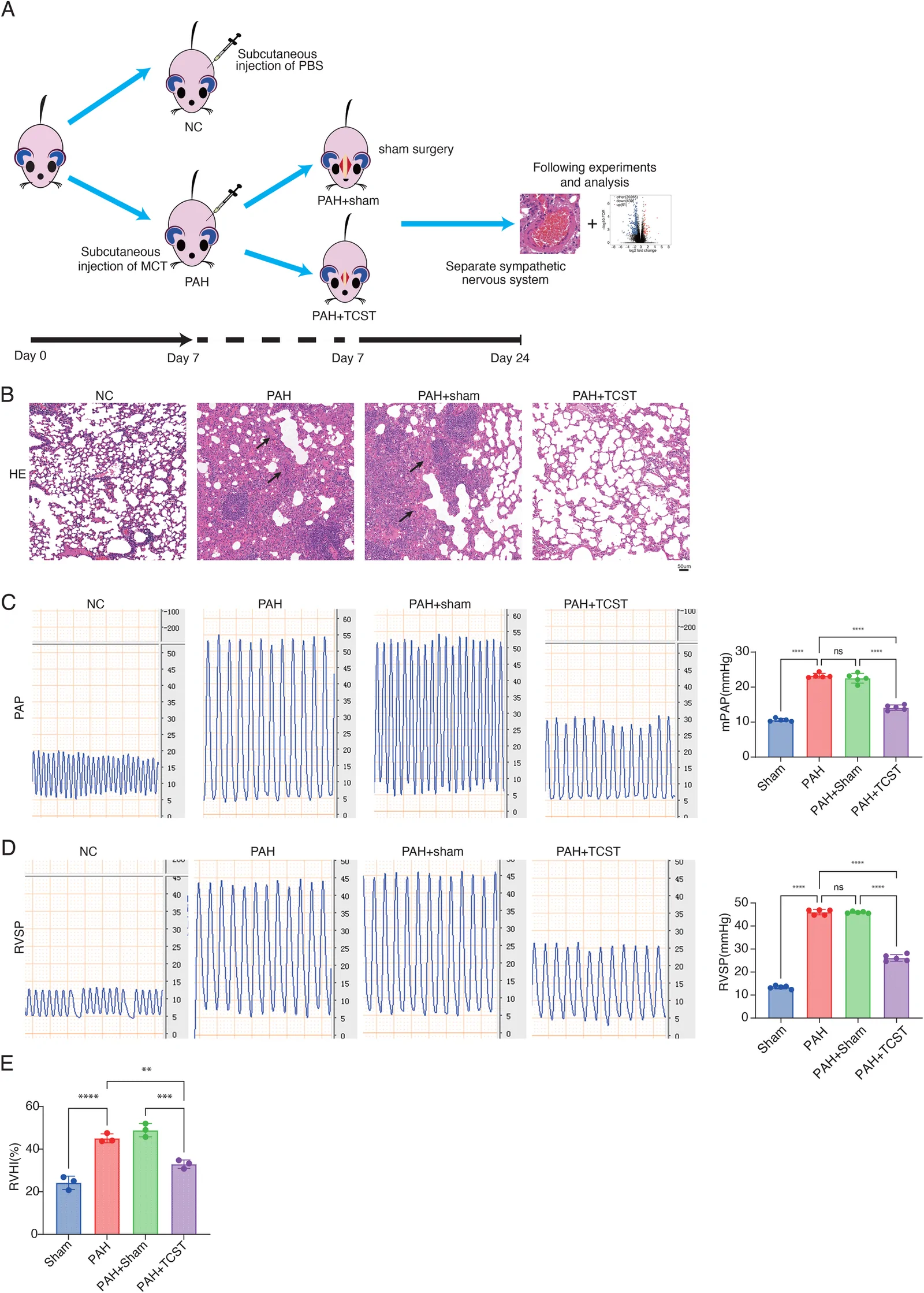
TCST ameliorates pulmonary pathology and hemodynamics in MCT-induced PAH rats. **(A)** Schematic diagram of an experimental workflow involving different treatment groups for PAH in mice. Normal Control (NC) mice received PBS; PAH was induced by MCT injection; PAH + Sham mice underwent sham surgery following MCT induction; and PAH + PSCT mice underwent transection of the cervical sympathetic trunk after MCT induction. **(B)** Representative H&E-stained lung sections from each experimental group, Scale ba*r* = 50*μ*m. The black arrows indicated hyper-proliferated endothelial cells. **(C)** Mean pulmonary arterial pressure (mPAP) measured 28 days post-MCT injection. **(D)** Right ventricular systolic pressure (RVSP) measured 28 days post-MCT injection. **(E)** Evaluation of the right ventricular hypertrophy index (RVHI) in MCT-induced PAH rats. Data are presented as mean with SD (*n* = 5 per group for C and D, *n* = 3 per group for E). **p < 0.01, ***p < 0.001, ****p < 0.001 (one-way ANOVA followed by Tukey's post hoctest).

### 2.3 Right ventricular systolic pressure (RVSP) measurement

Rats were anesthetized with pentobarbital sodium (40 mg/kg, i.p.) and placed in a supine position. A polyethylene catheter (PE-50) filled with heparinized saline was inserted into the right jugular vein and carefully advanced into the right ventricle. RVSP was recorded using a pressure transducer connected to the PE-50 catheter, and analyzed by a data acquisition system (MD3000, zhenghua biologic, China).

### 2.4 Mean pulmonary arterial pressure (mPAP) measurement

Following RVSP measurement, a left thoracotomy was performed to expose the heart and pulmonary artery. A PE-50 catheter was inserted into the main pulmonary artery through the right ventricular outflow tract, and mPAP was recorded using the same pressure transducer and data acquisition system.

### 2.5 Right ventricular hypertrophy index (RVHI) measurement

After hemodynamic measurements, hearts were excised via thoracotomy and washed with cold PBS. The right ventricle (RV) was carefully separated from the left ventricle plus septum (LV + SP). After blotting dry with filter paper, the RV and LV + SP tissues were weighed separately. RVHI was calculated using the following formula: RVHI=[RV/(LV + SP)] × 100%.

### 2.6 Euthanasia and anesthesia

For euthanasia, rats were intraperitoneally injected with 2.5% tribromoethanol at a dose of 50 μL/g body weight. For anesthesia during experimental procedures, rats were initially induced with 3%–5% isoflurane at an oxygen flow rate of 1 L/min, and subsequently maintained with 1%–2% isoflurane at an oxygen flow rate of 1 L/min.

### 2.7 Tissue processing

Following animal euthanasia, intact pathological tissues were collected and immediately fixed in 4% paraformaldehyde (PFA) for 48 h at 4 °C. After fixation, tissues were dehydrated through a graded ethanol series (70%, 80%, 95%, and 100%), cleared in xylene, and embedded in paraffin wax. Paraffin blocks were sectioned at 4 μm thickness using a microtome (KD-2258, KEDI, China). Sections were mounted on glass slides and dried at 65 °C for 2 h.

### 2.8 Hematoxylin and eosin (H&E) staining

Paraffin-embedded sections were deparaffinized in xylene (two changes, 20 min each) and rehydrated through a graded ethanol series (100%, 95%, 80%, 70%, and distilled water, 5 min each). Sections were stained with hematoxylin for 6 min, rinsed in distilled water, differentiated in 0.3% hydrochloric acid alcohol for 10 s, and blued in PBS for 5 min. Subsequently, sections were stained with eosin for 5 min, dehydrated through graded ethanol, cleared in xylene, and mounted with neutral resin. Images were captured using a whole-slide scanner (3DHISTECH, Hungary). Pulmonary vascular remodeling was assessed by measuring the medial wall thickness of small pulmonary arteries (50–100 μm in diameter) using ImageJ software.

### 2.9 RNA extraction and sequencing

Total RNA was isolated using TRIZOL reagent (15596-018, Ambion, USA) according to the manufacturer's protocol. RNA quality and concentration were assessed using an Ultrafine spectrophotometer (N50 Touch, IMPLEN, Germany), and RNA integrity was confirmed by 1.0% agarose gel electrophoresisFor each sample, 1 μg of total RNA was treated with RQ1 DNase (M6101, Promega, USA) to eliminate residual genomic DNA.

RNA sequencing was conducted for PAH_Sham and PAH_TCST groups with three biological replicates by Wuhan Ruixing Biotec Co., Ltd (http://www.rxbio.cc). For each sample, 1 μg of total RNA was treated with RQ1 DNase (M6101, Promega, USA) to remove residual DNA. Strand-specific RNA-seq libraries were constructed using the VAHTS® Universal V8 RNA-seq Library Prep Kit for Illumina (NR605, Vazyme, China). mRNA was enriched with VAHTS mRNA Capture Beads (N401, Vazyme, China), followed by fragmentation and synthesis of double-stranded cDNA. After end repair, A-tailing, and ligation to VAHTS RNA Multiplex Oligos Set 1 for Illumina (N323, Vazyme, China), the ligated products were amplified, purified, quantified and stored at −80 °C until sequencing. The use of dUTP marking during second-strand synthesis enabled strand-specific sequencing.

Libraries were sequenced on the Illumina NovaSeq Xplus platform following the manufacturer's instructions, generating 150 bp paired-end and 10G reads (100x) per sample.

### 2.10 RNA-seq data processing and alignment

Raw reads containing more than two undefined bases (N) were discarded. Adapter sequences and low-quality bases were trimmed using FASTX-Toolkit (v0.0.13). Reads shorter than 16 nt after trimming were also removed. The filtered reads were aligned to the gencode_mRatBN7.2 reference genome using HISAT2 (34) with an allowance of four mismatches. Uniquely mapped reads were used to quantify gene expression levels, reported as FPKM (fragments per kilobase of transcript per million fragments mapped) (35).

### 2.11 Differentially expressed genes (DEGs) analysis

We used uniquely mapped reads for raw read count quantification at the gene level by HTSeq (version 2.0.2). Raw counts, but not FPKM values, were used for DEGs analysis by DESeq2 package (36) from R software, whereas FPKM was used only for visualization of gene expression. Benjamini-Hochberg (BH) false discovery rate (FDR) correction with adjusted *P*-value (FDR) < 0.05, combined with |log_2_fold change| > 1 as the cut-off for DEGs identification.

### 2.12 Functional enrichment analysis

To sort out functional categories of DEGs, Gene Ontology (GO) functional enrichment and KEGG pathways analyses were performed using the KOBAS 2.0 server (37). Enrichment was defined using a hypergeometric test with Benjamini-Hochberg false discovery rate (FDR) correction (FDR < 0.05).

### 2.13 Prediction of transcription factors binding to targets

The 1377 transcription factors (TFs) of rat were retrieved from the AnimalTFDB4 database (https://guolab.wchscu.cn/AnimalTFDB4/#/Download). TFs with potential binding sites in the promoter regions of *Adra1d* and *Tacr1* were predicted using the Gene Transcription Regulation Database (GTRD) with default parameters (38). The promoter region (-2,000bp to +200 bp relative to the transcription start site) for each gene was queried in the GTRD to compile a list of all TFs with supported binding evidence by ChIP-seq. To focus on biologically relevant regulators, this list of potential TFs was then cross-referenced with the differentially expressed genes (DEGs) identified from our RNA-seq data. TFs presented in both the GTRD-predicted binding list and the DEGs were considered as high-confidence candidate regulators (olp-TFs) of target genes.

### 2.14 Co-expression analysis

Meanwhile, co-expression analysis was conducted between olp-TFs and the target genes, Adra1d and Tacr1, by Pearson's correlation coefficient (PCC) calculation. The TF-target pairs were kept for future analysis with criteria: absolute PCC value (|r|) ≥ 0.9 and *p*-value < 0.05.

### 2.15 Quantitative real-time PCR (qPCR)

RNA Extraction and Quality Control: Total RNAs from three biological replicates were extracted from lung tissues using TRIzol reagent according to the manufacturer's instructions. RNA concentration and purity were measured spectrophotometrically (NanoDrop). RNA integrity was confirmed by agarose gel electrophoresis. cDNA Synthesis: 1 µg of total RNA was reverse-transcribed into first-strand cDNA using a PrimeScript™ RT Reagent Kit with gDNA Eraser, following the supplier's protocol. qPCR Amplification: qPCR reactions were performed in triplicate using SYBR Green Premix on a StepOnePlus Real-Time PCR System. The reaction mix (20 µL) contained 10 µL SYBR Green Mix, 0.4 µM of each primer, 2 µL cDNA template, and RNase-free water. Cycling conditions: 95 °C for 30 s, followed by 40 cycles of 95 °C for 5 s and 60 °C for 30 s. Melt-curve analysis verified amplification specificity. Data Analysis: Each biological replicate contained three technical replicates. Relative gene expression was calculated using the 2^−ΔΔCt^ method (39), with *Gapdh* as the endogenous control. The primer sequences used in the study are shown in Table 1.

**Table 1:** Primer sequences for RT-qPCR experiment.

Gene	Sequences
*Foxa3*-F:	ATGTCCATCGCAGCATCCG
*Foxa3*-R:	CATACCGTGAAGGCAGTTAGAC
*Plcb1*-F:	GCCGTGTAATTCAGTGTTCAA
*Plcb1*-R:	ACAGTGTACAAGGGCAACATT
*Tacr1*-F:	GTGACATCCGTGAACATCCTC
*Tacr1*-R:	TCAACAAGGCTGGCAGAAGT
*Adra1d*-F:	GGGAACATACAGTTAGGAGTGT
*Adra1d*-R:	GAGGAAGTCAATGGACCTACC
*Gapdh*-F	TGCCACTCAGAAGACTGTGG
*Gapdh*-R	TTCAGCTCTGGGATGACCTT

### 2.16 ELISA

Sample Preparation: Lung tissue homogenates were prepared in cold PBS, centrifuged (10,000 g, 15 min, 4 °C), and supernatants were collected. Protein concentration was determined using a BCA Protein Assay Kit. Assay Procedure: IP3 levels were quantified using a commercial ELISA kit (E-EL-0059, Elabscience) according to the manufacturer's instructions. Briefly, 50 µL of sample or standard was added to pre-coated wells, followed by 50 µL of detection antibody. After 1 h incubation at 37 °C, wells were washed, and 100 µL of HRP-conjugated secondary antibody was added for 30 min. Following another wash, 90 µL of TMB substrate was added and incubated for 15 min in the dark. The reaction was stopped with 50 µL stop solution. Measurement and Calculation: Absorbance was measured at 450 nm using a microplate reader. IP3 concentrations were interpolated from the standard curve and normalized to total protein content.

### 2.17 Western blotting (WB) experiment

The samples were lysed in ice-cold RIPA Buffer (PR20001, Proteintech, China) supplemented with a protease inhibitor cocktail (4693116001, sigma, USA) and incubate on ice for 30 min. Samples were boiled for 45 min in 37℃ water with protein loading buffer (P1040, Solarbio, China) and loaded onto 10% SDS-PAGE gel and transferred onto 0.45 mm PVDF membranes (ISEQ00010, Millipore, USA). The PVDF membranes were then blocked for 1 h at room temprature and incubated overnight at 4℃ with primary antibody respectively against FOXA3 (anti-FOXA3, 1:10,000, antibody produced in mouse, PB0797, BOSTER, China) and GAPDH (1:10,000, antibody produced in mouse, 60004-1-Ig, Proteintech, China), followed by an incubation with horseradish peroxidase-conjugated secondary antibody (anti-rabbit, 1:10,000, SA00001-2, Proteintech, China) for 45 min at room temperature. Then, membranes were visualized using the enhanced ECL reagent (P0018FM, Beyotime, China) through chemiluminescence.

### 2.18 Alternative splicing analysis

The alternative splicing events (ASEs) and regulated alternative splicing events (RASEs) between TCST and Sham samples were defined and quantified by using the ABLas pipeline (version 1.0) as described previously (40). In brief, ABLas detection of ten types of ASEs was based on the splice junction reads, including exon skipping (ES), alternative 5' splice site (A5SS), alternative 3'splice site (A3SS), mutually exclusive exons (MXE), mutually exclusive 5'UTRs (5pMXE), mutually exclusive 3'UTRs (3pMXE), cassette exon, A3SS&ES and A5SS&ES. The AS ratio was calculated based on the junction reads between model and alternative splicing events. For differential analysis, Student's *t*-test was used to calculate the differential significance for the identified ASEs, which were defined as RASEs with false discovery rate cutoff of 5%.

For correlation analysis between RBPs and RASEs, expression levels of RBPs and AS ratios of RASEs were correlated by PCC method. Significant correlation pairs between RBPs and RASEs were selected with absolute PCC value (|r|) ≥ 0.9 and *p*-value < 0.05.

### 2.19 Statistical analysis

All statistical analyses were performed using R software (v4.0.5). The statistical figures were generated by ggplot2 package from R software. Data are presented as mean ± standard deviation (SD) from at least three independent experiments. Comparisons among multiple groups were performed using one-way analysis of variance (ANOVA) followed by Tukey's *post hoc* test. Comparisons between two groups were performed using Student's *t*-test. A *p*-value < 0.05 was considered statistically significant.

## 3 Results

### 3.1 TCST alleviates pulmonary arterial hypertension in MCT-induced PAH rat

To investigate whether TCST mitigates PAH pathology, we established a PAH model in rats using MCT and performed TCST (Figure 1A). Successful TCST was confirmed by the onset of Horner's syndrome on the ipsilateral side, presenting as enophthalmos, miosis and ptosis. Compared with the negative control (NC) group, rats in the PAH and PAH + sham groups exhibited significant alveolar structural damage, marked thickening of the pulmonary interstitium and small pulmonary arterial walls, luminal stenosis, disrupted endothelial cell continuity and medial smooth muscle cell proliferation and hypertrophy with disorganized arrangement. Most alveolar structures were effaced. In contrast, the PAH + TCST group showed notable improvements in these pathological features, including relatively preserved alveolar architecture and slight alveolar dilation in some regions (Figure 1B). Hemodynamically, mPAP (Figure 1C) and right ventricular systolic pressure (RVSP) (Figure 1D) were significantly elevated in the PAH and PAH + sham groups compared to the NC group. However, both mPAP (Figure 1C) and RVSP (Figure 1D) were significantly reduced in the PAH + TCST group, and the extent of right ventricular hypertrophy was also ameliorated (Figure 1E). These findings collectively demonstrate that TCST effectively alleviates the symptoms of PAH in the rat model.

### 3.2 TCST modulates gene expression to attenuate PAH through inhibition of inflammation and calcium-driven proliferation

To elucidate the molecular mechanisms underlying the therapeutic effect of TCST, we performed RNA-seq on lung tissues from the PAH + sham and PAH + TCST groups. Principal component analysis revealed that TCST induced substantial gene expression profile changes between these two groups (Figure 2A). Comparative analysis identified 500 differentially expressed genes (DEGs) in the PAH + TCST group *vs.* the PAH + sham group, including 61 up-regulated and 439 down-regulated genes (Figure 2B). To further investigate the functional roles of the DEGs, GO and KEGG enrichment analyses were performed. For the up-regulated DEGs, GO analysis revealed enrichment in molecular functions such as protein binding, RNA polymerase II cis-regulatory region sequence-specific DNA binding, as well as biological processes including inflammatory response, immune response, apoptotic response, and positive regulation of apoptosis. KEGG pathway analysis showed that up-regulated genes were enriched in the cytokine-cytokine receptor interaction signaling pathway (Figure 2C). Regarding the down-regulated DEGs, GO enrichment analysis identified molecular functions such as dynein chain binding, calcium ion binding, calcium-dependent protein binding, ATP binding, ATP hydrolysis activity, and growth factor activity. Biological processes included adaptive immune response, positive regulation of calcium ion concentration, and positive regulation of cell proliferation. KEGG pathway analysis further revealed that down-regulated genes were enriched in pathways such as the complement cascade, cytokine-cytokine receptor interaction, primary immunodeficiency, calcium signaling pathway, PPAR signaling pathway, AMPK signaling pathway, and PI3K-Akt signaling pathway (Figure 2D). Collectively, these transcriptomic data suggest that TCST exerts its therapeutic effects in PAH by modulating multiple biological processes, with a particular emphasis on suppressing calcium signaling and cell proliferation pathways, while also influencing inflammatory and immune-related pathways. These findings provide a molecular framework for further investigating the key regulators and downstream targets underlying TCST-mediated amelioration of PAH.

**Figure 2:**
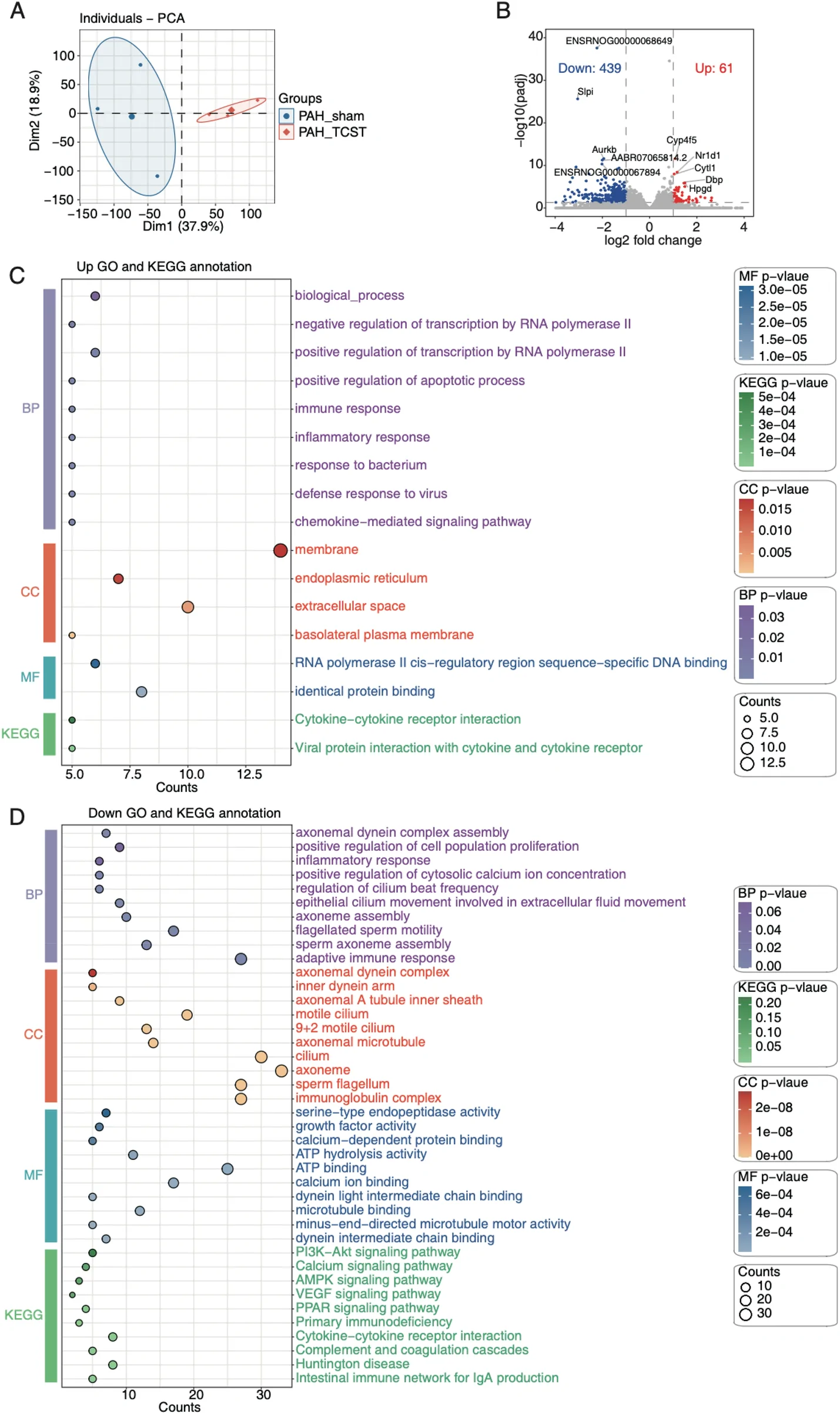
TCST modulates the lung transcriptome in PAH rats. **(A)** Principal component analysis (PCA) of transcriptomic profiles after normalization of gene expression levels. The confidence ellipse is shown for each experimental group (*n* = 3 per group). **(B)** Volcano plot displaying the distribution of differentially expressed genes (DEGs) between PAH + TCST and PAH + sham groups; Top five up and down DEGs were labeled. **(C)** Gene Ontology (GO) and Kyoto Encyclopedia of Genes and Genomes (KEGG) pathway enrichment analysis (41–43) of up-regulated DEGs. **(D)** GO and KEGG pathway enrichment analysis (41–43) of down-regulated DEGs.

### 3.3 TCST remodels the transcription factor regulatory landscape in PAH

To elucidate the transcriptional regulatory landscape underlying the therapeutic effects of TCST, we intersected the differentially expressed genes (DEGs) with a curated transcription factor (TF) dataset. This analysis identified 15 differentially expressed transcription factors (DETFs) that were significantly regulated following TCST intervention (Figure 3A). Among them, *Zbtb16*, *Nkx2-8*, *Klf2*, *Nr1d1*, *Dbp*, and *Zfp683* were significantly upregulated, whereas *Bhlha15*, *Tcf24*, *Barx2*, *Pax9*, *Foxa3*, *Nr1i2*, *Tbxt*, *Elf3*, and *AABR07030823.1* were downregulated (Figure 3B). To further uncover the downstream biological processes regulated by TFs following TCST, we performed Gene Ontology (GO) enrichment analysis on genes co-expressed with the 15 DETFs. The co-expressed genes were significantly enriched in biological processes including “inflammatory response,” “positive regulation of cell population proliferation,” “positive regulation of cytosolic calcium ion concentration,” and “calcium-mediated signaling” (Figure 3C). These results suggest that TCST may coordinately regulate multiple PAH-associated pathological pathways, including inflammatory response, cell proliferation, and calcium signaling, through reprogramming of TF expression. To further dissect the core genes involved in regulating these pathways, we constructed a regulatory network linking functional modules to their target genes (Figure 3D). The network revealed that genes involved in the inflammatory response pathway also connected to calcium signaling and proliferation modules, as exemplified by Cxcl13 and Bmp2. Notably, the expression of six genes with established pro-PAH roles—*Bmp2*, *Ptgs2*, *Cxcl13, Wnt7b*, *Tacr1*, and *Adra1d*—was significantly downregulated following TCST intervention (Figure 3E).

**Figure 3:**
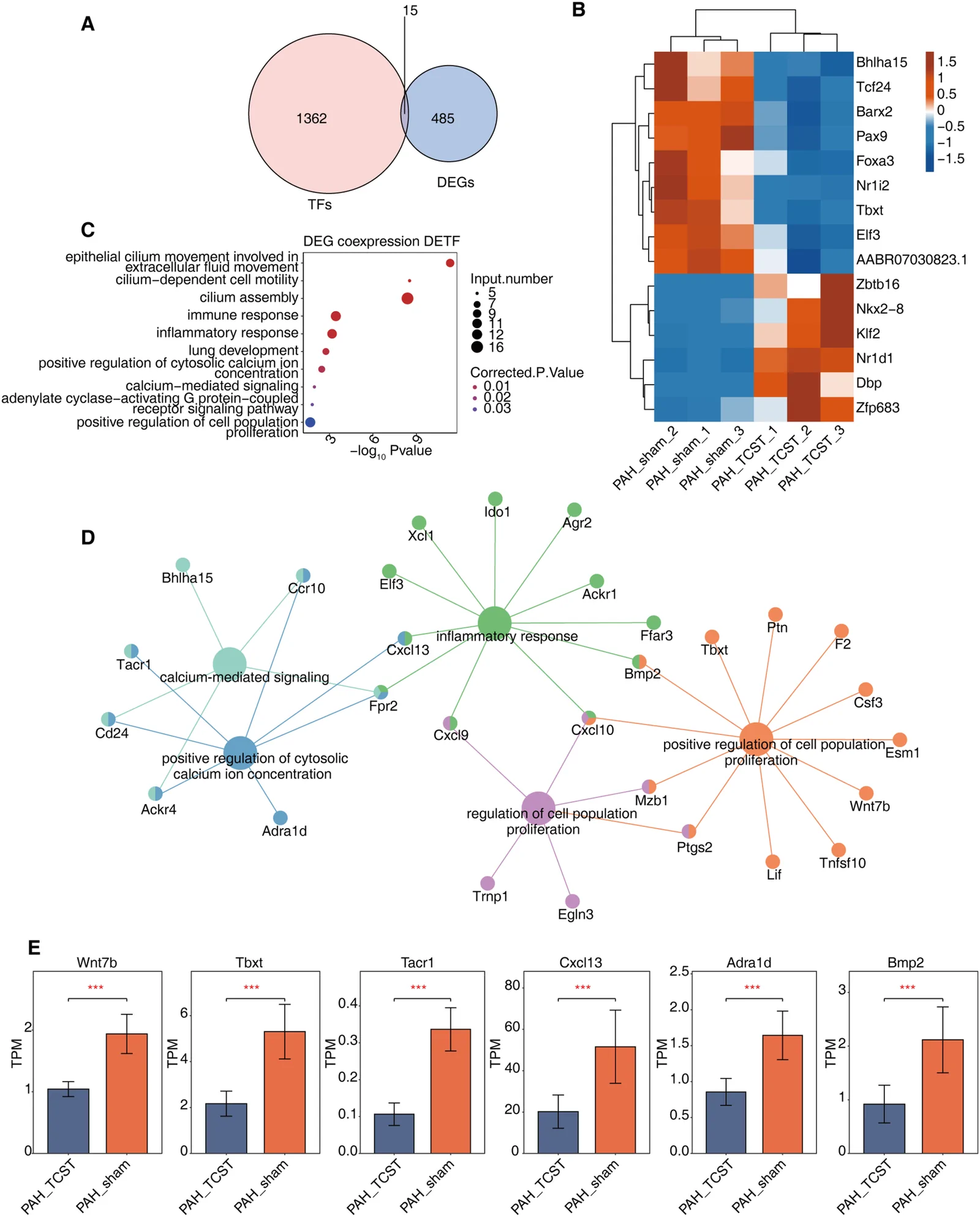
Identification of differentially expressed transcription factors and their co-expressed functional modules. **(A)** Venn diagram showing the overlap between differentially expressed genes (DEGs) and transcription factors (TFs). **(B)** Heatmap of 15 differentially expressed transcription factors (DETFs) between PAH + sham and PAH + TCST groups. **(C)** Gene Ontology (GO) enrichment analysis of genes co-expressed with the 15 DETFs **(D)** Network diagram illustrating the relationships between enriched GO terms and their co-expressed target genes, categorized into functional modules including inflammatory response, cell proliferation, and calcium signaling. E) Expression levels of six representative hub genes (Bmp2, Ptgs2, Cxcl13, Wnt7b, Tacr1, and Adra1d) derived from RNA-Seq data. Data are presented as mean with SD (*n* = 3 per group for RNA-Seq). *p < 0.05, **p < 0.01, ***p < 0.001.

### 3.4 TCST suppresses calcium signaling pathway activation

Given that the GO enrichment analysis identified “positive regulation of cytosolic calcium ion concentration” and “calcium-mediated signaling” as key targets of the TF co-expression network (Figure 3C), and that calcium signaling pathway genes including *Adra1d* and *Tacr1* were significantly downregulated following TCST (Figure 3E), we further validated the activation status of the calcium signaling pathway. To explore the upstream regulators of *Adra1d* and *Tacr1*, we screened for transcription factors with predicted binding activity to their promoter regions using the GTRD database. Intersecting these candidates with the downregulated DEGs identified from our RNA-seq data revealed *Foxa3* as a common transcription factor potentially regulating both *Adra1d* and *Tacr1* (Figure 4A). To further validate this regulatory relationship, we used the JASPAR database to obtain the DNA-binding motif of FOXA3 and applied the FIMO tool to predict putative binding sites within the promoter regions of *Adra1d* and *Tacr1*. Multiple potential binding sites for this motif (GTAAACA) were identified in the promoters of both genes (Figure 4B). Co-expression analysis further substantiated a strong positive correlation between *Foxa3* expression and both *Adra1d* (r = 0.96, *p* = 0.002) and *Tacr1* (r = 0.95, *p* = 0.004) (Figure 4C). We further verified the key molecules at the transcriptional and protein levels using qPCR and ELISA techniques. qPCR results showed that, compared with the PAH + sham group, the mRNA expression levels of *Foxa3* and its downstream target genes *Adra1d* and *Tacr1* in the lung tissues of the PAH + TCST group were significantly downregulated (Figure 4D). Concurrently, the expression of *Plcb1*, encoding phospholipase C*β*1 (a key enzyme mediating IP3 production), was also significantly reduced (Figure 4E). Then we validated the decreased expression level of FOXA3 in PAH + TCST group at protein level (Figure 4F, Supplementary Figure S1). Since IP3 is a critical second messenger produced by PLC*β*1-mediated hydrolysis of PIP2, directly mediating calcium release from the endoplasmic reticulum, we further measured IP3 levels in lung tissues by ELISA. The results showed that IP3 concentration in the PAH + TCST group was significantly lower than that in the PAH + sham group (Figure 4G). Collectively, the RT-qPCR, ELISA, and WB validation results confirmed the transcriptomic finding that the calcium signaling pathway was suppressed following TCST. Together with the coordinated changes in inflammation and proliferation pathways observed in Figure 3, these functional validations further support the notion that TCST exerts its therapeutic effects through systemic reprogramming of transcription factor networks and coordinated suppression of multiple PAH-associated pathological pathways, with calcium signaling being one of the representative pathways effectively inhibited.

**Figure 4:**
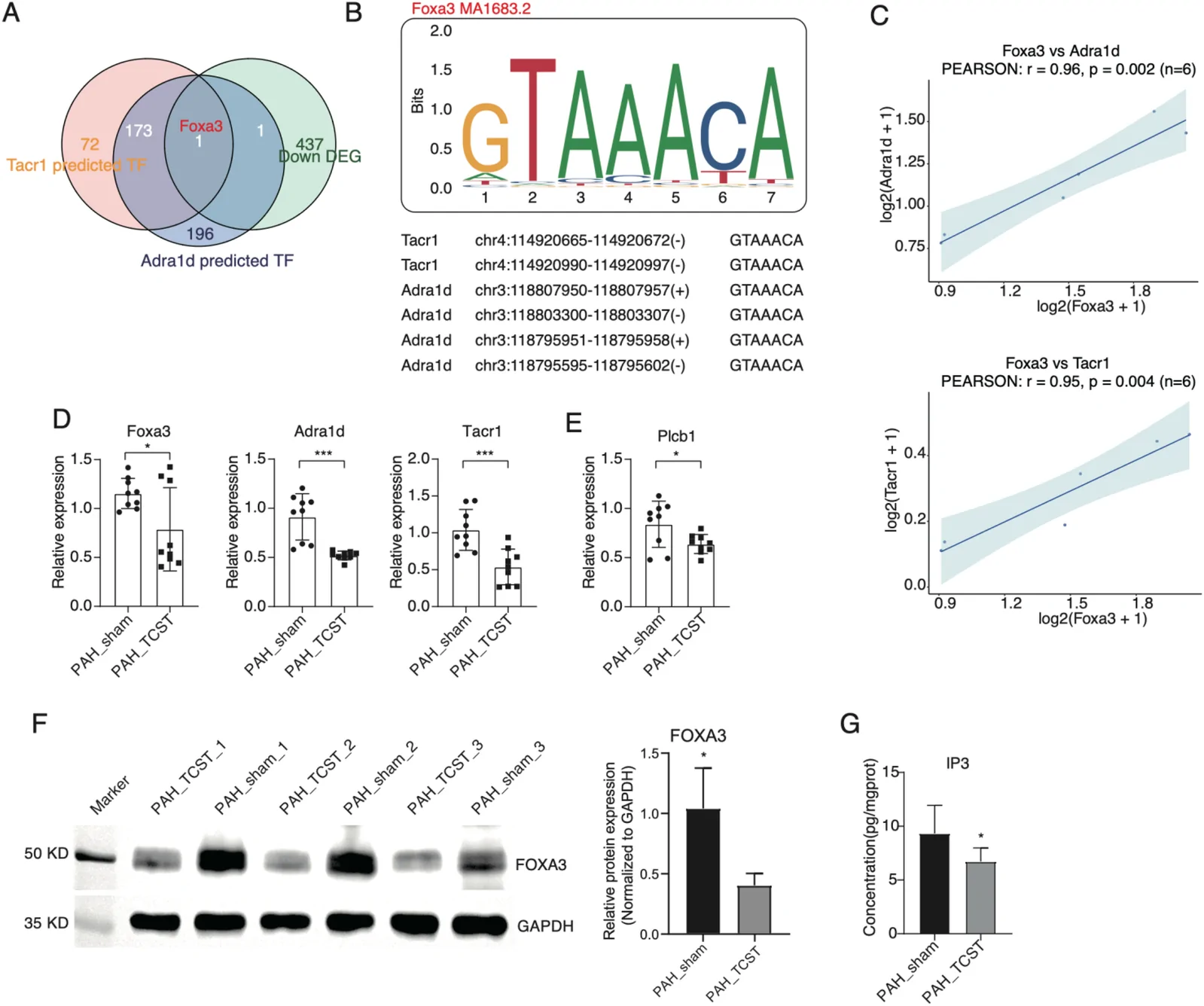
Validation of Foxa3-mediated calcium signaling pathway suppression by TCST. **(A)** Venn diagram illustrating the overlap between predicted transcription factors for *Adra1d* and *Tacr1* promoters using the GTRD database and down-regulated DEGs. **(B)** Schematic representation of predicted FOXA3 binding sites within the promoter regions of *Adra1d* and *Tacr1*, identified using the JASPAR and FIMO databases. **(C)** Scatter plots showing co-expression correlation between *Foxa3* and the target genes *Adra1d* and *Tacr1*. **(D)**-**(E)** Bar plot showing qPCR validation of *Foxa3*, *Adra1d*, *Tacr1*, and *Plcb1* expression levels across groups (*n* = 3 per group with three technical replicates). **(F)** WB result for FOXA3 in PAH + TCST and PAH + sham groups; Right panel was the statistical result (*n* = 3 per group). **(G)** Bar plot showing ELISA measurement of IP3 concentration across groups (*n* = 3 per group). Data are presented as mean with SD. * *p* < 0.05, ** *p* < 0.01, *** *p* < 0.001.

### 3.5 TCST modulates alternative splicing via RBP-mediated post-transcriptional regulation

To investigate whether TCST regulates gene expression at the post-transcriptional level, we performed comprehensive alternative splicing analysis using ABLas program on the RNA-seq data. Multiple types of ASEs were identified across all samples, including alternative 5’ splice sites (A5SS), alternative 3’ splice sites (A3SS), mutually exclusive exons (MXE), intron retention (IR), exon skipping (ES), cassette exon, and their combined events (A5SS&SE, A3SS&SE, 5pMXE, 3pMXE) (Figure 5A). Differential splicing analysis between the PAH + TCST and PAH + sham groups identified a substantial number of differentially alternative splicing (DAS) events, with intron retention (IR), A5SS, and A3SS events being the predominant types (Figure 5B). These findings indicated that TCST not only alters gene expression levels but also reprograms the splicing landscape of pulmonary transcripts. To understand the biological significance of TCST-modulated splicing events, we performed GO and KEGG enrichment analysis on genes harboring DAS events (Figure 5C). The enriched pathways included apoptosis, transcriptional regulation, cell adhesion, and fluid shear stress. Since RBPs are master regulators of alternative splicing, we further analyzed the differentially expressed RBPs co-expressed with DAS genes. A total of 14 RBPs were identified as differentially expressed following TCST, including *Zbtb16, Zfp683*, *Tent5c*, *Enpp3*, *Rab6b*, *Ttc21a*, and *Aicda*. We constructed a co-expression network linking these RBPs to their putative target genes that underwent alternative splicing (Figure 5D). The network revealed several RBP-DAS gene pairs with strong correlation coefficients, suggesting that TCST may regulate alternative splicing through modulating RBP expression. Notably, three PAH-relevant genes exhibited significant alternative splicing changes following TCST: *Aldh2* showed decreased A3SS, while *Il1rn* and *Foxm1* showed increased A5SS and A3SS, respectively. Together, these findings suggest that TCST may ameliorate PAH by modulating RBP expression to alter alternative splicing patterns of target genes at the post-transcriptional level.

**Figure 5:**
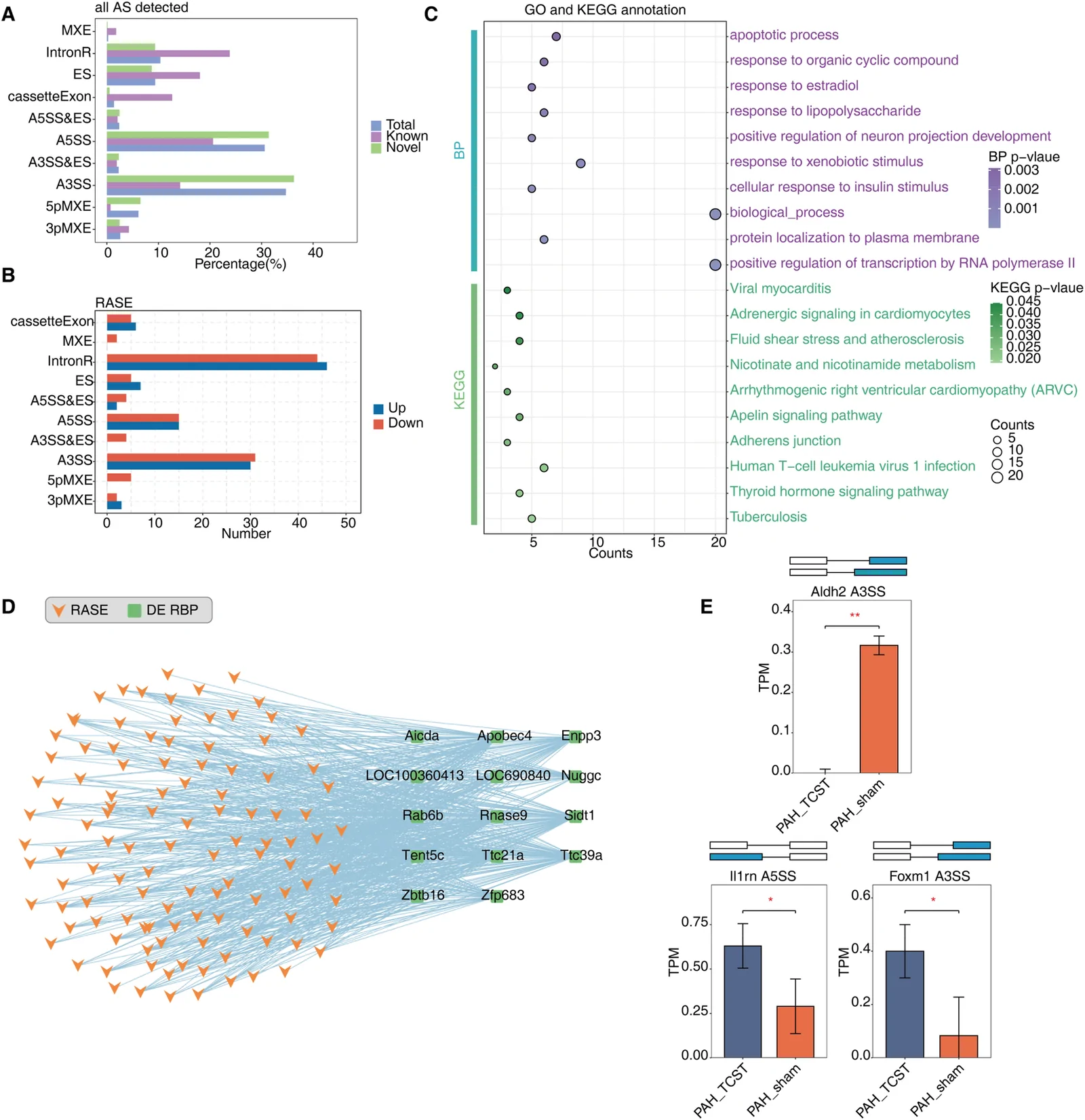
Alternative splicing analysis and RBP-mediated post-transcriptional regulation modulated by TCST. **(A)** Classification of alternative splicing event types identified by ABLas, including the dominant ES, A5SS, A3SS, MXE, and IR. **(B)** Summary of regulated alternative splicing events (RASEs) between PAH + sham and PAH + TCST groups. **(C)** GO/KEGG enrichment analysis of genes harboring DAS events. **(D)** Co-expression network of differentially expressed RNA-binding proteins (RBPs) and their associated DAS genes; node color indicates expression change direction (red: upregulated, blue: downregulated). **(E)** Schematic representation of alternative splicing patterns of three PAH-relevant genes (*Aldh2*, *Il1rn*, and *Foxm1*) following TCST, with exon inclusion levels shown as bar plots (*n* = 3 per group). Data are presented as mean with SD. * *p* < 0.05, ** *p* < 0.01.

## 4 Discussion

This study systematically evaluated the therapeutic potential of TCST in an MCT-induced rat model of PAH, and revealed its dual-layer molecular mechanism involving transcription factor network reprogramming and RBP-mediated alternative splicing regulation. Our major findings include: (1) TCST significantly attenuated pulmonary vascular remodeling and reduced mPAP, RVSP, and RVHI; (2) TCST reshaped a co-expression network of differentially expressed transcription factors, with their target genes enriched in functional modules including inflammation, proliferation, and calcium signaling; (3) TCST modulated the expression of multiple RBPs and the alternative splicing patterns of their downstream target genes; (4) TCST suppressed the calcium signaling pathway through the Foxa3/Adra1d-Tacr1 axis, with reduced IP3 levels. Together, these findings provide new insights into the molecular mechanisms by which TCST ameliorates PAH.

Transcription factors serve as core molecular switches linking extracellular signals to gene expression. This study provides the first systematic delineation of the bidirectional regulatory landscape of differentially expressed transcription factors in a TCST-treated PAH model, suggesting selective reprogramming of the transcriptional network by TCST. GO enrichment analysis revealed that genes co-expressed with DETFs were significantly enriched in PAH-relevant functional modules, including inflammatory response, cell proliferation, and calcium signaling. Furthermore, multiple PAH-associated genes (Bmp2, Ptgs2, Cxcl13, Wnt7b, Tacr1, and Adra1d) were consistently downregulated following TCST. The BMP2/BMPRII axis is generally considered protective in the pulmonary vasculature, exerting antiproliferative effects in PASMCs through the PPAR*γ*/apoE pathway (44). In the MCT-PAH model, impaired BMPRII signaling is a well-documented feature (45). Therefore, the downregulation of Bmp2 observed after TCST should not be interpreted in isolation as a direct therapeutic benefit. Rather, it likely reflects a broader remodeling of the BMP signaling landscape in the pulmonary vascular microenvironment, and its functional significance can only be assessed in conjunction with BMPR2 receptor expression and downstream Smad1/5/8 activity. COX-2 (encoded by Ptgs2) promotes PASMC proliferation through an increased TXA₂/PGI₂ ratio (46); Wnt7b drives pulmonary artery smooth muscle precursor proliferation via the Wnt/*β*-catenin-PDGFR axis and is highly expressed in PAH patient lung tissues (47); CXCL13 is elevated in both pulmonary vascular lesions and serum of IPAH patients, correlating with pulmonary vascular remodeling (48). TACR1 and ADRA1D are key factors in the calcium signaling pathway. ADRA1D has been identified as an important regulator of PAH (49), is significantly upregulated in PAH rat models, and promotes PASMC proliferation through activation of the ERK-1/2 pathway (50). TACR1 encodes NK1R, the receptor for substance P, which mediates elevated pulmonary arterial pressure; NK1R antagonists or substance P depletion can prevent PAH development (51), and RNAi-mediated silencing of Tacr1 or treatment with NK1 receptor antagonists attenuates pulmonary hypertension and right ventricular hypertrophy in MCT-induced PAH rat models (52). The coordinated downregulation of these genes—which are involved in pro-proliferative, pro-inflammatory, or pro-contractile processes—suggests that TCST may attenuate the deleterious crosstalk among proliferative signals, inflammatory microenvironment, and vasoconstrictive drivers through systemic suppression of multiple PAH-associated transcriptional programs, thereby exerting a broad-spectrum pathological network blocking effect at the transcriptional level. Meanwhile, qPCR and ELISA validation confirmed that TCST significantly suppressed the expression of Foxa3, Adra1d, Tacr1, and Plcb1, and reduced IP3 production. Given that aberrant calcium signaling is a core driver of PASMC proliferation and vasoconstriction in PAH (4, 8, 53, 54), and that elevated cytosolic Ca^2^⁺ directly activates pro-inflammatory transcription factors including NF-*κ*B and NFAT (55), suppression of the calcium signaling pathway by TCST may exert synergistic effects on both proliferative and inflammatory pathways, collectively contributing to the amelioration of pulmonary vascular remodeling.

At the post-transcriptional level, the co-expression network of differentially expressed RBPs and DAS genes suggests that TCST may influence alternative splicing through modulation of RBP expression. Notably, Aldh2, Il1rn, and Foxm1 each exhibited significant splicing pattern changes following TCST: Aldh2 showed decreased A3SS, and its deficiency exacerbates hypoxia-induced PAH, increasing right ventricular systolic pressure and pulmonary vascular remodeling; ALDH2 regulates endothelial dysfunction and smooth muscle cell proliferation through the p38/NF-*κ*B/IL-6 pathway (56). Il1rn and Foxm1 showed increased A5SS and A3SS, respectively—the former encodes IL-1Ra, an endogenous antagonist of IL-1R1; IL-1R1 is significantly overexpressed in PAH patient lung tissues, and IL-1R1 knockout or treatment with recombinant IL-1Ra (Anakinra) significantly attenuates hypoxia-induced PAH, with IL-1β-stimulated PASMC proliferation being completely blocked by Anakinra (57). The latter encodes FOXM1, a pro-proliferative transcription factor regulated by FTO-mediated m⁶A modification; FTO expression is elevated in PAH smooth muscle cells and promotes proliferation through stabilizing Foxm1 mRNA, while smooth muscle-specific Fto knockout protects mice from hypoxia-induced PAH (58).

This study has several limitations that should be carefully considered when interpreting the results. First, the TF and RBP regulatory networks were constructed primarily based on bioinformatic predictions from RNA-seq data. Therefore, future studies are required to validate these findings through ChIP-qPCR, dual-luciferase reporter assays, RNA immunoprecipitation (RIP) or CLIP-seq methods. Second, the biological functions of PAH-related gene downregulation and the alternative splicing events need to be validated through gain- or loss-of-function experiments at the cellular or animal level to assess their impact on PASMC proliferation, migration, or inflammatory responses. Third, although the MCT-induced rat model is a classical PAH model, its pathophysiological mechanisms primarily rely on endothelial injury and inflammatory responses, which do not fully recapitulate the heterogeneity of human PAH. Therefore, whether the findings of this study are applicable to other PAH subtypes requires further validation in multiple animal models and clinical samples. Although Horner's sign confirmed successful TCST, we did not directly quantify sympathetic activity; thus, the association between the molecular effects of TCST and decreased sympathetic tone remains inferential, and future studies incorporating these parameters are needed. Forth, TCST faces significant challenges in clinical translation. In future studies, we plan to incorporate serial echocardiography including RV internal diameter at end-diastole (RVEDD) and end-systole (RVESD), to stage RV adaptation and to more rigorously validate the functional benefits of TCST, in conjunction with tissue-level markers such as norepinephrine and tyrosine hydroxylase to provide a more comprehensive evaluation of TCST-mediated sympathetic modulation. Future research should also explore minimally invasive or reversible sympathetic modulation strategies, such as ultrasound-guided repeated SGB, or reversible nerve blockade technologies, to facilitate clinical translation.

## 5 Conclusion

In summary, this study demonstrates that TCST effectively alleviates pulmonary vascular remodeling and hemodynamic abnormalities in the MCT-induced rat model of PAH. Mechanistically, TCST acts through selective reprogramming of transcription factor networks and regulation of RBP-mediated alternative splicing to coordinately modulate multiple PAH-associated pathways, including inflammation, proliferation, and calcium signaling, at both the transcriptional and post-transcriptional levels. These findings deepen our understanding of sympathetic nervous system involvement in PAH pathogenesis and provide a theoretical foundation for developing PAH treatment strategies based on neuroimmunomodulation.

## Data Availability

The datasets presented in this study can be found in online repositories. The names of the repository/repositories and accession number(s) can be found below: https://www.ncbi.nlm.nih.gov/geo/, GSE328273.
