# Supplementary material for: Transection of the cervical sympathetic trunk ameliorates pulmonary arterial hypertension by reprogramming transcription factor networks and alternative splicing in rat model
Source: Front Cardiovasc Med. 2026 Oct 28;13:1967847. doi: 10.3389/fcvm.2026.1967847 (PMC13752110; doi:10.3389/fcvm.2026.1967847)
Supplement: Supplementary file 1 [file Datasheet1.docx]

Supplementary Figures

**Figure S1. The original WB images for FOXA3 and GAPDH. The result of GAPDH contained a non-specific band.**
